# Br-Mediated Spin-State Control in Nickelocene and Cobaltocene

**DOI:** 10.1021/jacs.5c17873

**Published:** 2026-01-10

**Authors:** Donglin Li, Nan Cao, Adam S. Foster, Shigeki Kawai

**Affiliations:** † Center for Basic Research on Materials, National Institute for Materials Science, 1-2-1 Segen, Tsukuba, Ibaraki 305-0047, Japan; ‡ Department of Applied Physics, 174277 Aalto University, Espoo 11100, Finland; § Nano Life Science Institute (WPI-NanoLSI), Kanazawa University, Kakuma-machi, Kanazawa, Ishikawa 920-1192, Japan; ∥ Graduate School of Pure and Applied Sciences, University of Tsukuba, 1-1-1 Tenodai, Tsukuba, Ibaraki 305-8571, Japan

## Abstract

Single-molecule magnets represent promising materials due to their stable magnetic states and long relaxation times. Precise engineering of their quantum properties is of importance to realize advanced electronic devices, such as high-density data storage, quantum computing, and spintronics. Here, we investigate the spin state of nickelocene (NiCp_2_) and cobaltocene (CoCp_2_) molecules manipulated by Br atoms. With a combination of scanning tunneling microscopy and density functional theory calculations, we reveal that the high electronegativity of Br atoms significantly changes the magnetic properties of both NiCp_2_ and CoCp_2_. For NiCp_2_, the spin-state transition from its intrinsic *S* = 1 to *S* = 1/2 occurs when the Br atoms underlying the molecule consist of more than five atoms. The spin state is further shifted to *S* = 0 by approaching a Br-terminated tip toward the molecule. In contrast, a strong hybridization between CoCp_2_ and Br atoms leads to a complete quenching of its spin moment. This strategy for tuning molecular spin states provides a promising route toward the scalable design of molecular spintronic devices.

## Graphical abstract

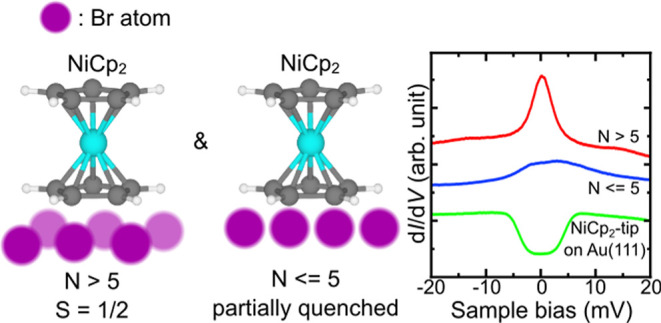

## Introduction

Single-molecule magnets (SMMs) have emerged as a fascinating frontier in nanoscience and materials research, drawing attention due to their remarkable potential in high-density data storage,^1–3^ quantum computing,^4,5^ spintronics,^6–9^ and the realization of qubits.^10,11^ These unique systems possess stable magnetic states together with long magnetic relaxation times in the absence of a magnetic field, offering an unprecedented platform for manipulating magnetic properties. The magnetism in SMMs results from the unpaired d or f electrons of magnetic atoms embedded within the molecules. These electrons are responsible for a distinctive spin state that can be further tuned by various parameters such as chemical architecture of surrounding ligands^12–14^ and mechanical forces.^15^ The ability to precisely engineer these quantum properties offers possibilities in the design of advanced electronic devices where each molecule behaves as an individual information unit or quantum bit for the next generation of quantum computing and data storage technologies.

Recent advancements in scanning tunneling microscopy (STM) have established it as a powerful tool in the study of SMMs, offering unprecedented precision in monitoring, characterizing, and even manipulating their magnetic properties at the individual molecule level.^16–23^ When combined with inelastic electron tunneling spectroscopy (IETS), we can obtain not only spatially resolved images but also detailed spectroscopic insights into the energy levels and spin excitations within these systems.^24,25^ The high spatial and energy resolution allows investigation of molecular magnetism and the quantum behavior of spin states. For instance, Ormaza et al. demonstrated a reversible switching between spin 1 and 2 with a NiCp_2_ molecule by varying the tip–molecule gap from tunneling to the contact regime using STM.^26^ Similarly, Vegliante et al. tuned the spin state of a polycyclic aromatic hydrocarbon molecule by manipulating the molecular conformation using the STM tip while simultaneously detecting changes in the spin excitation spectrum.^27^ These pioneering studies highlight the invaluable capability of these STM-based techniques to probe and control molecular spin states at the atomic scale. However, achieving systematic and reliable strategies for tuning the spin state of organometallic complexes through their interaction with the surroundings remains to be systematically addressed.

In this study, we focus on the spin state modulations in NiCp_2_ and CoCp_2_ molecules as prototypical molecular magnets by incorporating Br buffer atoms between them and the Au(111) substrate (Scheme 1). We use low-temperature STM and IETS to demonstrate that the underlying Br atoms play a decisive role in modulating the spin states of NiCp_2_ and that tip-induced perturbations offer an additional avenue of control. When five or fewer Br atoms are involved, the spin state changes gradually, whereas the presence of more than five Br atoms drives a switch to *S* = 1/2. In contrast to a metal tip, the spin state is completely quenched by approaching it with a Br-terminated tip. Similarly, CoCp_2_ exhibits such strong interaction with Br atoms that its spin state is always quenched regardless of the number of Br atoms. Our density functional theory (DFT) calculations provide microscopic insights into the mechanisms behind these spin state modifications. The calculations reveal that Br adsorption induces significant charge transfer from NiCp_2_ to the Br layer, accompanied by a downward shift and splitting of the Ni 3d orbitals, which stabilizes the *S* = 1/2 states. In contrast, CoCp_2_ exhibits a much stronger hybridization with Br, leading to complete quenching of its spin density. Moreover, simulations of the tip–molecule junction show that additional charge redistributions and enhanced hybridization as the Br-tip approaches drive the spin state of NiCp_2_ from *S* = 1/2 toward a fully quenched *S* = 0 state, consistent with our experimental observations. These results establish a clear correlation between the electronic structure modifications and the experimentally observed spin-state transitions. Our approach not only deepens our understanding of spin regulation at the molecular level but also paves the way for developing more scalable strategies for spin control in future spintronic applications.

**Scheme 1:**
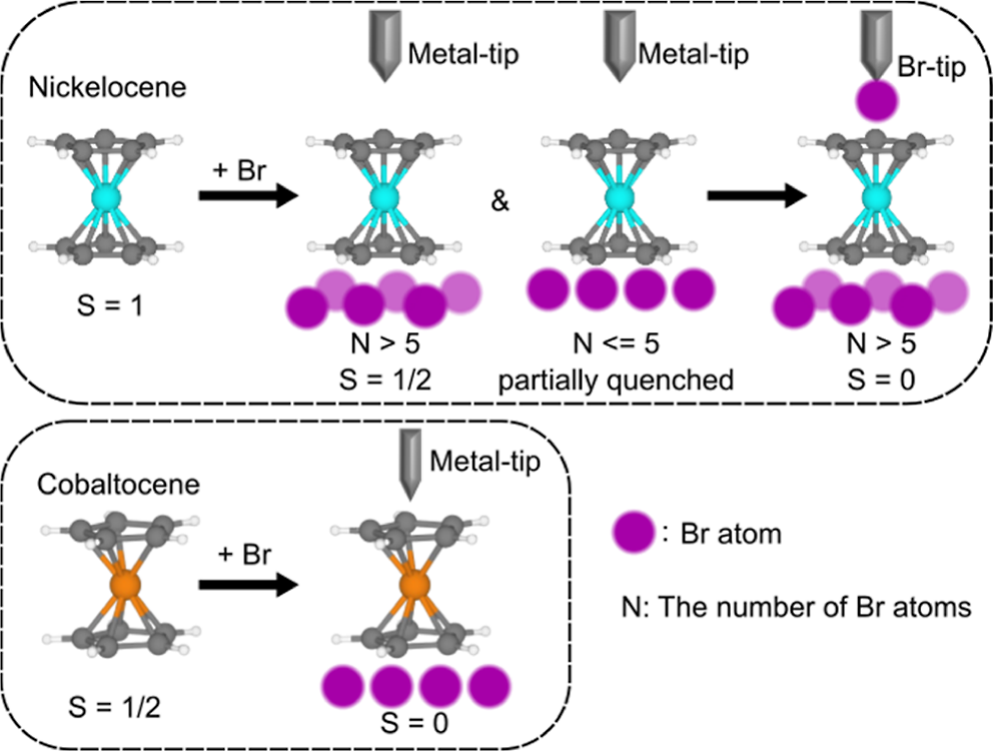
Schematic Illustration of NiCp_2_ and CoCp_2_ Spin States Modulated by Incorporating Br Atoms on the Surface and the Tip^a^

a Atom colors: white, gray, blue, orange, and purple atoms correspond to H, C, Ni, Co, and Br atoms, respectively. The apparent sizes of Br atoms and NiCp_2_ molecules in the scheme correspond to their electronic states rather than their geometric dimensions.

## Results and Discussion

After NiCp_2_ molecules and Br atoms were deposited onto a clean Au(111) surface, isolated NiCp_2_ molecules were observed at 4.3 K (Figure 1a). These molecules appear as disk-shaped contrast patterns in STM images, consistent with a flat lying adsorption geometry with the top cyclopentadienyl (Cp) ring exposed to vacuum. This adsorption configuration agrees with those on bare Au(111), as reported in the previous work.^28^ At given tunneling conditions, two distinct types of NiCp_2_ molecules were observed (Figure 1b,c): Type 1 appears as isolated molecules at a glance with an asymmetric contrast, similar to that of tilted NiCp_2_ molecules,^29,30^ while Type 2 is surrounded by Br atoms and is likely adsorbed on them. To verify this, line profiles taken across the molecules (Figure S1a) were analyzed, showing that both types are tilted. In addition, both exhibit similar apparent heights of approximately 480 pm, which is ∼50 pm higher than that of NiCp_2_ adsorbed directly on bare Au(111) (430 pm, Figure S1b). This height difference is comparable to the apparent STM contrast of individual Br atoms on Au(111) (Figure S1c), suggesting that the NiCp_2_ molecules are adsorbed on the Br atoms.

**Figure 1:**
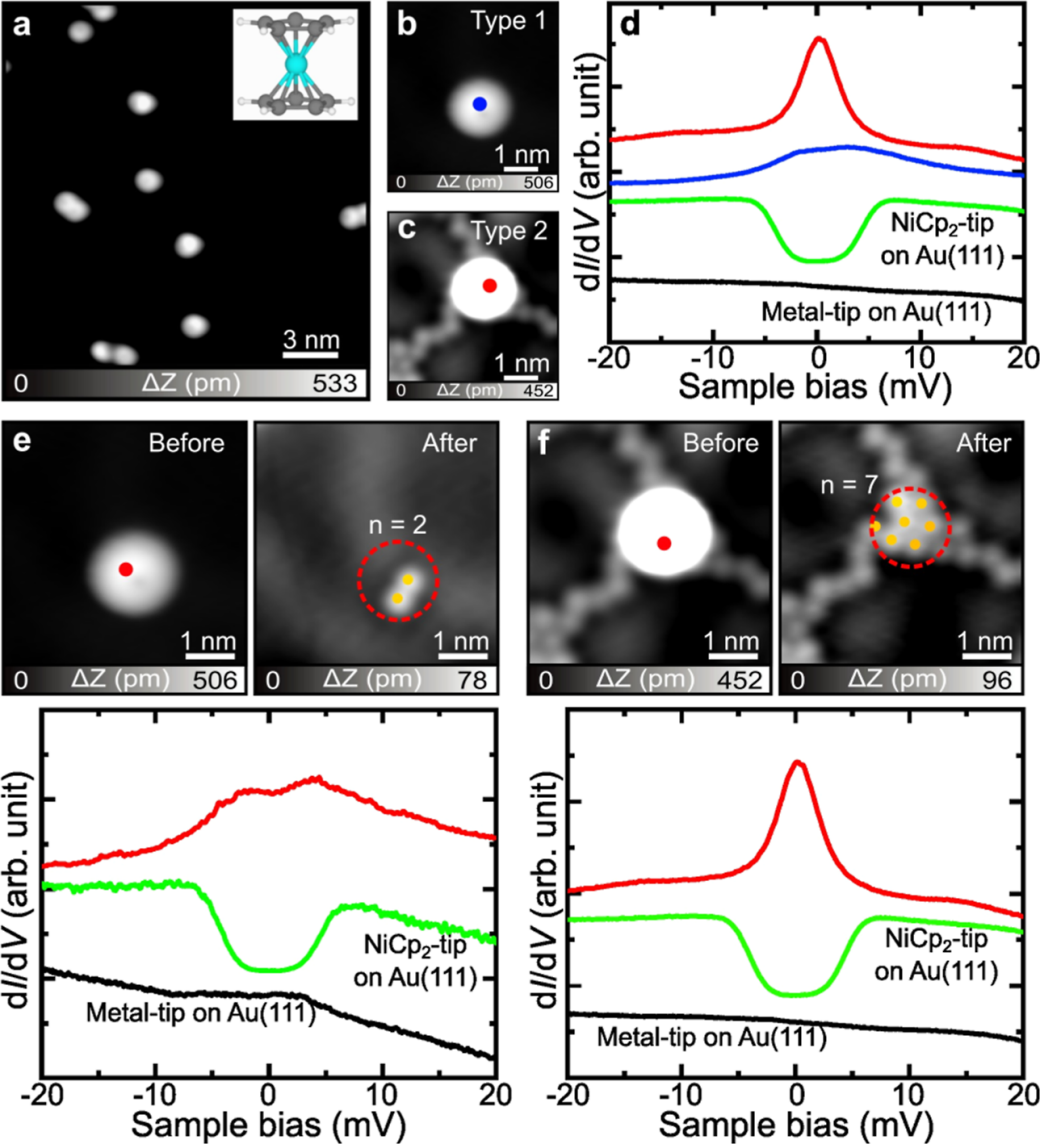
Effect of different numbers of Br atoms on the spin state of a NiCp_2_ molecule. (a) STM image of the NiCp_2_ molecule adsorbed on Br clusters. The inset shows the chemical structure of the NiCp_2_ molecule. (b, c) Close-up STM images of Type 1 and Type 2 NiCp_2_, respectively. The contrast in (c) is adjusted to highlight the surrounding Br atoms. (d) d*I*/d*V* spectra recorded at the marked sites in (b) and (c) using a metal tip. The green curve is acquired on a clean Au(111) surface with a NiCp_2_ tip. (e) STM images taken before and after picking up Type 1 from the surface. The dotted red circle indicates its original position. Orange dots indicate Br atoms. d*I*/d*V* spectra at a lower panel recorded at the site marked by a red dot, using a metal tip. (f) STM images taken before and after picking up Type 2 from the surface. The dotted red circle indicates its original position. The d*I*/d*V* spectrum at a lower panel recorded at the site marked by a red dot, using a metal tip. Green curves in (e, f) were obtained from a clean Au(111) surface with a NiCp_2_ tip. Scanning parameters: (a, b, c, e, f) *V* = 0.2 V, *I* = 10 pA; (d) *V*_ac_ = 0.5 mV. The black d*I*/d*V* curves were recorded on the bare Au(111) surface using a metal tip.

To probe the spin states of Type 1 and Type 2 NiCp_2_ molecules, STS measurements were performed near the Fermi energy (Figure 1d). As a reference, we first examined a single NiCp_2_ adsorbent on a bare Au(111) surface using a metallic tip (the green curve). The corresponding differential conductance (d*I*/d*V*) spectrum shows typical symmetric step-like features, attributed to inelastic spin-flip excitations of an *S* = 1 system.^25^ In contrast, the d*I*/d*V* spectra of Type 1 and Type 2 exhibit significantly different features: Type 1 shows a broadened feature near the Fermi energy (*E*_F_) (blue curve). In contrast, Type 2 exhibits a sharp zero-bias peak (red curve), which is characteristic of a Kondo resonance. The different spectral shapes indicate distinct spin states. The zero-bias resonance observed in Type 2 reflects the presence of a localized *S* = 1/2 spin screened by conduction electrons—the Kondo effect. To determine the number of Br atoms underlying the molecule, NiCp_2_ molecules were removed from the surface by approaching the STM tip at 1 mV until a sudden change in the tunneling current was detected. Consequently, the NiCp_2_ molecule was attached to the tip. The successful attachment of the NiCp_2_ molecule to the tip was confirmed by symmetric spin-flip features in the d*I*/d*V* spectrum measured with the tip on bare Au(111).^28^ After manipulation, two Br atoms appeared beneath Type 1 NiCp_2_ (Figure 1e), whereas seven Br atoms were observed beneath Type 2 NiCp_2_ (Figure 1f). These observations suggest a correlation between the number of underlying Br atoms and the spin states of NiCp_2_ molecules. To further investigate the influence of the underlying Br atoms on the spin, we performed a series of manipulations of NiCp_2_ molecules (Figure S2). It was found that when five or fewer Br atoms are present underneath the molecule, only broadened d*I*/d*V* spectral features are observed, whereas six or more Br atoms lead to the emergence of a pronounced zero-bias resonance. Notably, Br prefer to form quasi-(√3×√3) packing structures (Figure S3).^31^ Although the manipulation may slightly modify the local packing structure of Br atoms, this does not affect the counting results. This trend suggests a spin-state transition from *S* = 1 to 1/2, modulated by the local Br environment. This transition is likely driven by Br-induced charge transfer, which alters the electronic structure of NiCp_2_ and consequently its spin state.

To further investigate influences of the underlying Br atoms to the spin state of NiCp_2_, extended Br islands were formed on Au(111), as all NiCp_2_ molecules were found to adsorb exclusively on the Br atoms (Figure 2a). The d*I*/d*V* spectrum acquired with a metal tip above different NiCp_2_ molecules on Br islands consistently exhibits zero-bias resonances flanked by symmetric side peaks (blue, red, and green curves in Figure 2b). This spectral character closely resembles that previously observed for CoCp_2_ molecules (*S* = 1/2).^24,32^ In line with the spin-state transition inferred from Figures 1 and S2, the zero-bias peak observed on Br islands is characteristic of a Kondo resonance arising from a localized *S* = 1/2 spin state. The high-resolution STM image of an individual NiCp_2_ molecule on Br islands (Figure 2c) reveals slight asymmetry in its appearance, likely due to a tilt of the molecular axis relative to the surface normal. Moreover, the spatial mapping of the Kondo resonance shows a two-lobe pattern (Figure 2d). To reproduce this feature, we modeled the NiCp_2_ molecule adsorbed on a Br island on Au(111), considering several adsorption geometries (Figure S4). The corresponding simulated d*I*/d*V* map using conf2 (Figure S4) in Figure 2e is in good agreement with the experimental image in Figure 2d. Moreover, the computed magnetic moment of the molecule is about 0.9 μ_B_, corresponding to an effective spin state of *S* = 1/2. This agreement provides further support that the Br environment stabilizes a localized *S* = 1/2 spin in NiCp_2_. The side peaks marked by black lines in Figure 2b are ascribed to the vibrational excitations of the molecule. Additionally, these peaks show little dependence on the adsorption configuration, although their energies vary somewhat between molecules. Furthermore, the typical symmetric steps observed in the d*I*/d*V* spectra after transferring NiCp_2_ onto a metal tip confirm that the NiCp_2_ molecule remains intact during the process.

**Figure 2:**
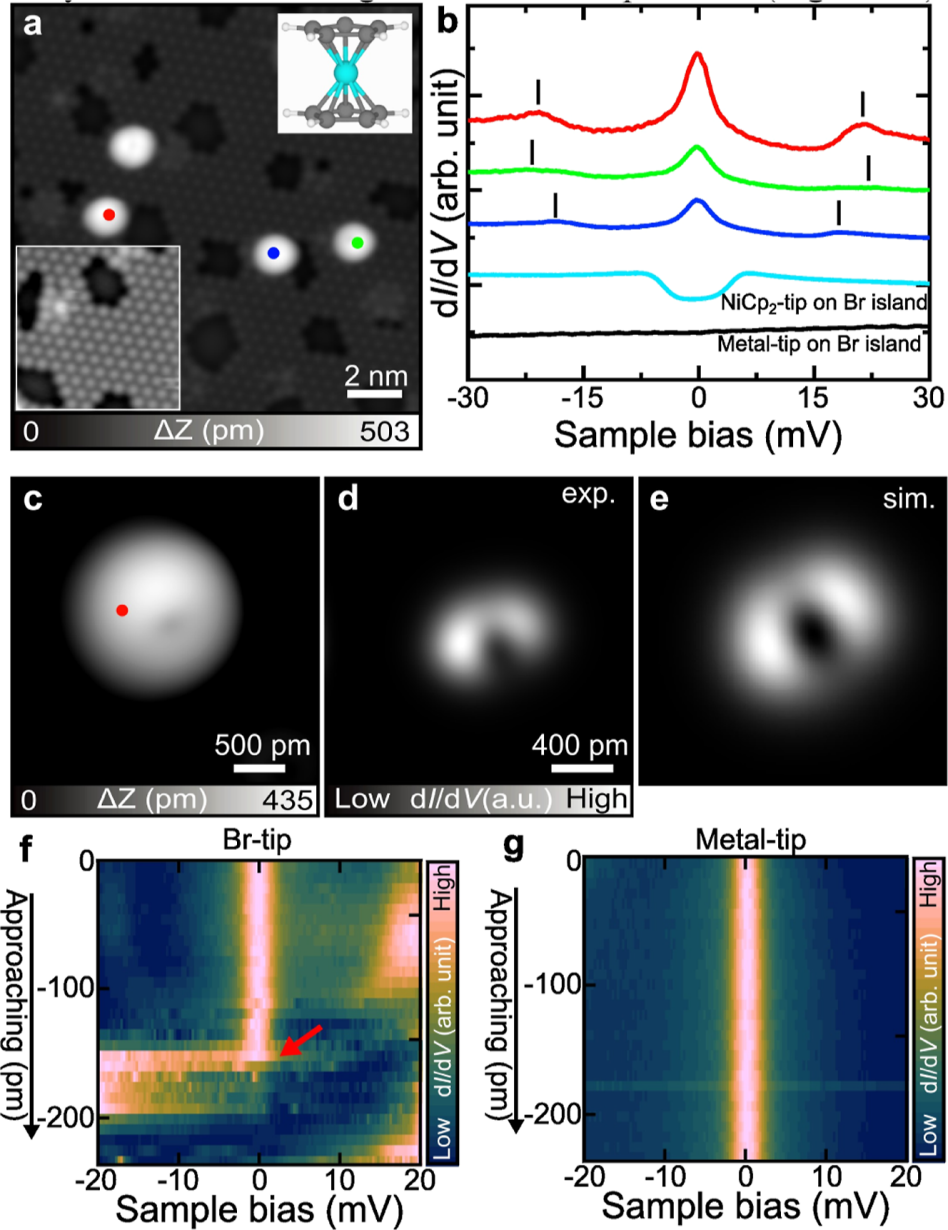
Magnetic properties of NiCp_2_ molecules adsorbed on a Br island. (a) STM image of NiCp_2_ molecules adsorbed on Br islands. The inset shows the corresponding area with adjusted contrast to highlight the Br island. (b) d*I*/d*V* spectra recorded at the sites marked in (a). The green and black curves were acquired over the Br island using a NiCp_2_-tip and a metal tip, respectively. (c,d) Close-up views of an STM image of a NiCp_2_ adsorbed on the Br island and its corresponding constant-height d*I*/d*V* image acquired with a metal tip at a sample bias of 1 mV. (e) Simulated spin distribution of a NiCp_2_ adsorbed on the Br island (image sizes: 12 × 12 Å^2^). (f, g) Two-dimensional intensity plots of d*I*/d*V* spectra acquired on the NiCp_2_ with a Br-tip (f) and a metal tip (g) at 240 pm and different tip–sample distances. The intensities have been normalized. The initial set point was *V* = 20 mV, *I* = 300 pA. Measurement parameters: *V* = 0.2 V, *I* = 10 pA in (a, b); *V*_ac_ = 0.5 mV in (b, f, g).

To further modulate the spin state of NiCp_2_, we attempted to attach an additional Br atom directly to the top of the molecule. However, it was technically challenging to precisely position a Br atom at a specific site of NiCp_2_. As an alternative, we used a Br-terminated tip. The Br atom was transferred to the tip apex by gradually approaching the tip toward a Br atom on the surface until a sudden change in tunneling current was detected.^33^ With the Br-terminated tip, we performed height-dependent STS measurements on the NiCp_2_ molecule adsorbed on the Br island (Figure 2f). Interestingly, as the tip approached, we observed an abrupt disappearance of the Kondo resonance, indicating quenching of the 1/2 spin S state. Importantly, this switching is reversible as the *S* = 1/2 state recovers once the tip is retracted. This behavior suggests a controllable spin-state transition from *S* = 1/2 to *S* = 0, triggered by the presence of the Br atom at the tip apex. For comparison, identical height-dependent STS measurements were performed using a metal tip (Figure 2g). In this case, no significant variation of the Kondo resonance was observed with a reduction in the tip–sample distance, thereby confirming that the quenching effect is specific to the presence of the Br atom at the tip apex. Notably, the side peaks corresponding to molecular vibrational excitations observed in Figure 2f (around 20 mV) are not visible in Figure 2g, possibly due to the different tip conditions affecting the vibrational energy. A clearer comparison, based on the representative curves extracted from Figure 2f,g, is shown in Figure S5. These results collectively demonstrate that the local environment introduced by the Br-terminated tip can reversibly modulate the spin state of NiCp_2_ molecules within the Au surface–Au tip junction. The observed quenching of the Kondo resonance suggests a modification of the local electronic environment upon the Br-tip approach. In addition, we performed systematic studies using iodine­(I) atoms and found that electronegativity plays a key role in modulating the molecular spin state (Figure S6).

To elucidate the experimentally observed spin-state transitions, we carried out DFT calculations on a NiCp_2_ molecule adsorbed on Br-decorated Au(111) surfaces. Geometry optimizations reveal that the adsorption configuration remains largely similar for adsorption on different numbers of Br atoms. The molecule adopts a slightly asymmetric configuration, tilting by ∼5–15° relative to the surface plane (Figure 3a). However, the computed magnetic moment decreases systematically as the number of underlying Br atoms increases (Figure 3b). As a reference, the gas-phase NiCp_2_ molecule carries a total moment of 1.7 μ_B_ (Ni atom: 1.3 μ_B_) in our DFT calculations, consistent with an *S* = 1 state (comparison of using different functionals is listed in Table S1, and DFT + U tests are shown in Table S2 and Figure S7). Upon adsorption on a single Br atom, the molecular and Ni magnetic moments drop to 1.25 μ_B_ and 0.8 μ_B_, respectively. With three Br atoms, these values further decrease to 1.04 μ_B_ and 0.68 μ_B_, respectively, and eventually stabilize at ∼0.87 μ_B_ (molecule) and ∼0.56 μ_B_ (Ni) when the molecule is on the 9-atom Br island on Au(111) (conf3, Figure S8). Since Br and Au atoms are nonmagnetic in all configurations, the reduction of the total moment by roughly half upon adsorption on a Br island suggests the molecular spin changes from *S* = 1 to *S* = 1/2. This spin transition is in agreement with the spectroscopic fingerprints observed experimentally. Furthermore, various molecular adsorption configurations on the surface were examined, and the magnetic moments are in the range of 0.87–0.95 μ_B_ (Figure S8 and Table S3). The pronounced sensitivity to the number of underlying Br atoms indicates that the spin-state transition is not primarily driven by structural rearrangement but rather originates from electronic interactions at the molecule–substrate interface.

**Figure 3:**
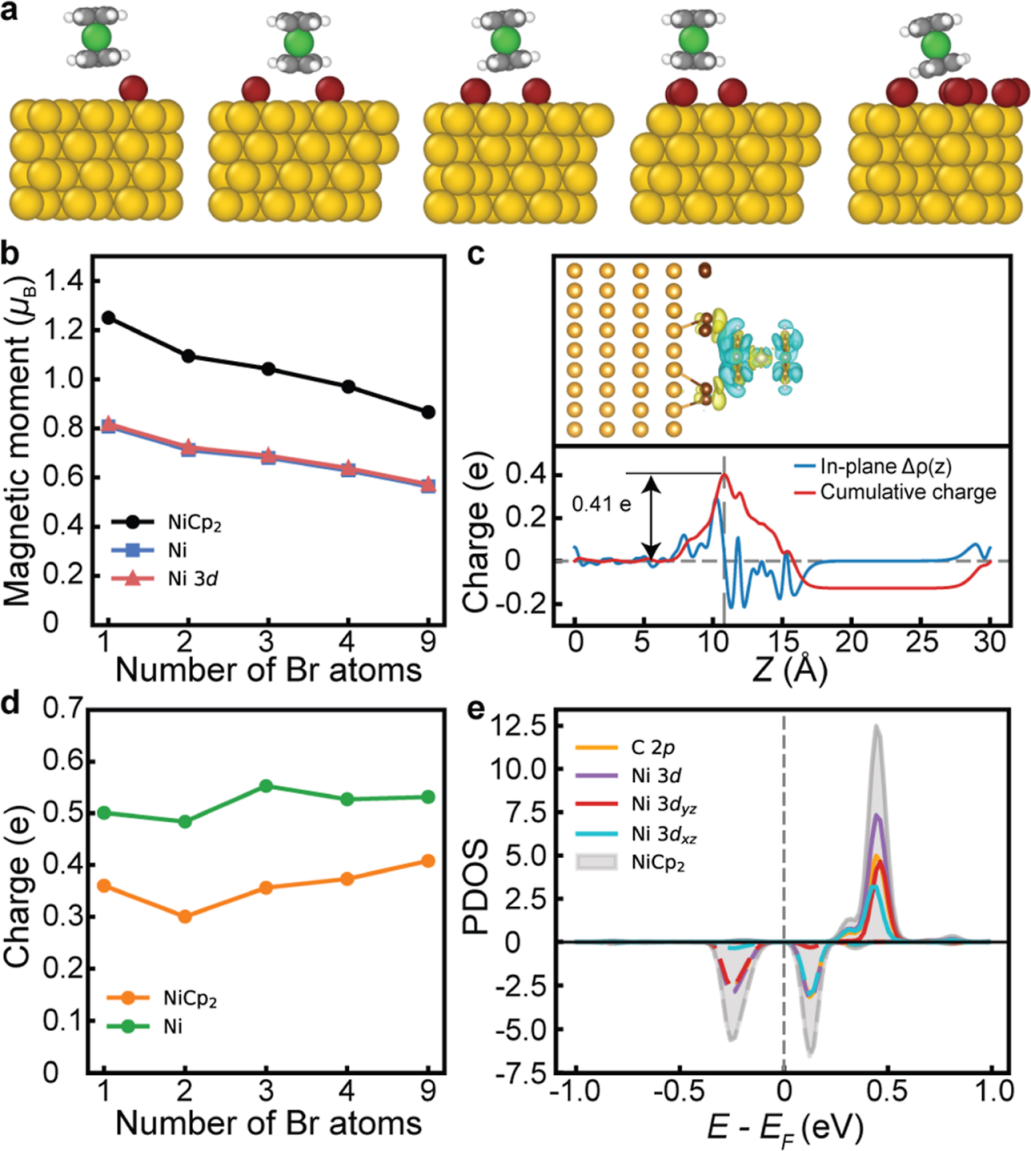
Computed structural and electronic properties of NiCp_2_ molecules adsorbed on different numbers of bromine atoms on Au(111). (a) Energetically favored adsorption geometries of NiCp_2_ molecules on one, two, three, four, and nine Br atoms from right to left. (b) Computed magnetic moments of NiCp_2_ molecules, Ni atoms, and Ni 3d orbitals in adsorption configurations in (a). (c) The upper panel shows the side view of the NiCp_2_ on the Br island (9 atoms) on Au(111), showing charge transfer from the molecule to the Br island. The cyan contour represents the regions of charge depletion, while the yellow contour represents the charge accumulation (isosurface at 0.0003e/Å^3^). The charge density difference is defined as Δρ = ρ­(total) – ρ­(molecule) – ρ­(Br/Au), where ρ­(total) is the total charge density, ρ­(molecule) is the charge density of NiCp_2_, and ρ­(Br/Au) is the charge density of the Br island and Au(111) substrate. The lower panel is the DFT-calculated in-plane electron density difference plot. The blue curve represents the in-plane charge density difference, and the red curve is the integrated in-plane charge density difference along the *z* direction. The results show ∼0.41e is cumulated between the molecule and the Br island, comparable with the amount of charge obtained from Bader charge analysis. (d) The net charge of NiCp_2_ molecules in various adsorption configurations in (a), derived from Bader charge analysis. (e) Projected density of states (PDOS) for the adsorption on the Br island in (c). The original degenerate Ni 3d_*yz*_ and 3d_*xz*_ orbitals split upon adsorption compared with the gas-phase molecule (see details in Figure S8a). The red and cyan curves near the Fermi level display a singly occupied molecular orbital and a singly unoccupied molecular orbital, respectively.

We first quantified the charge transfer associated with the spin-state transition for NiCp_2_ adsorbed on a Br island on Au(111). The planar-averaged charge density difference analysis shows an electron depletion of about 0.4 e from the molecule to the Br island (Figure 3c), similar to previous reports in which standing sandwich-type molecules exhibit charge delocalization toward the Au(111) surface.^34,35^ This value is consistent with our Bader charge analysis (0.41 e, Figure 3d). This loss of electron density correlates directly with the reduction of the molecular magnetic moment from 1.7 μ_B_ in the gas phase to 0.87 μ_B_ on Br/Au(111). Although Br atoms and NiCp_2_ molecules are comparable in geometric size, a series of calculations with increasing Br converge (from one to four atoms) confirm that the net charge transfer increases from 0.3 to 0.4 e, in line with the progressive reduction of the magnetic moment (Figure 3b). This trend is likely due to the reduced interaction between the extended delocalized π orbitals of NiCp_2_ and the Au(111) substrate at a higher Br coverage. The charge density depletion mainly involves the Ni 3d-dominated frontier orbitals. Consequently, one of the components becomes depleted, while the other remains singly occupied, yielding an *S* = 1/2 state.

From a molecular orbital perspective, the spin-polarized PDOS clarifies the transition (Figure 3e). In the gas phase (Figure S8), the frontier molecular orbitals originate from π-symmetric hybridization between the Ni 3d_*xz*_ and 3d_*yz*_ orbitals and the p orbitals of the Cp rings. These orbitals are degenerate; their majority-spin components distribute below *E*_F_ (occupied), while the minority-spin components distribute above the *E*_F_ (unoccupied). This configuration gives two parallel unpaired electrons and a total magnetic moment of 1.7 μ_B_ (*S* = 1). When the molecule is adsorbed onto the Br island on Au(111), the degeneracy of the 3d_*xz*_/3d_*yz*_ orbitals is lifted, accompanied by a charge transfer of about 0.4 e from the molecule to the Br island. Specifically, the 3d_*xz*_-dominated orbital shifts upward in energy and becomes unoccupied, while the 3d_*yz*_-dominated orbital remains singly occupied. As a result, only one unpaired electron is retained, and the molecule adopts an *S* = 1/2 spin state. Compared with the gas phase, the minority-spin PDOS shifts closer to the *E*_F_ and the exchange splitting is reduced, consistent with weak interfacial hybridization on Br/Au(111). This interpretation is supported by a detailed spin-resolved PDOS and molecular orbital analysis in Figure S9, which shows that the frontier states become spin-asymmetric hybrid orbitals and that spin density is redistributed toward the Br island. Together, these analyses provide the microscopic origin of the spin-state transition from *S* = 1 to S = 1/2.

To clarify the Br-tip-induced quenching of the spin *S* = 1/2 state, we modeled the tip by placing a Br atom atop a Au atom on a Au(111) plane (Figure 4a). The Br-tip was positioned above a NiCp_2_ molecule, and the full junction was relaxed at three different vertical distances *z* = 15.3, 14.9, and 14.3 Å. As the tip approaches, the total molecular magnetic moment progressively decreases from 0.7 to 0.62 to 0.47 μ_B_ (Ni: from 0.45 to 0.39 to 0.3 μ_B_, Figure 4b). Meanwhile, the net molecular charge evolves from +0.38 to +0.23 to −0.07 e (Figure 4c), suggesting that the initial electron depletion is gradually compensated. Within the experimentally relevant distance range (15.3 Å–14.9 Å), the Br-tip retains weakly negative and the total charge on the Br island is essentially unchanged, whereas the charge depletion of the Au substrate is slightly increased (Table S4 and Figure S10). At the shortest distance (14.3 Å), the compressed junction geometry induces a more pronounced local redistribution of charge, consistent with the strong mechanical perturbation in this configuration (Figure S11). Overall, these results indicate that the tip does not cause substantial charge transfer but rather induces charge redistribution and interfacial polarization across the junction.

**Figure 4:**
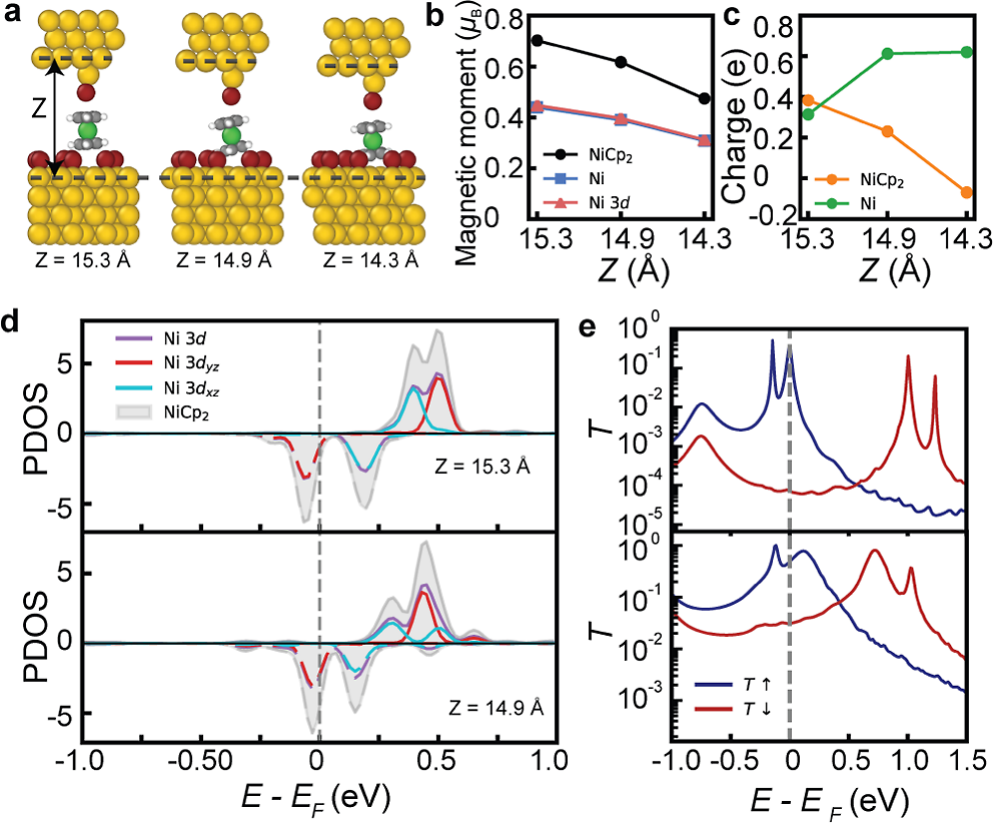
Computed electronic and magnetic properties of the molecular junction. (a) Structural configurations of the calculated molecular junction, with progressively decreasing the distance *z* between the tip surface and the substrate surface from right to left. (b) Computed magnetic moments of NiCp_2_ molecules, Ni atoms, and Ni 3d orbitals in molecular junctions in (a). (c) The net charge of NiCp_2_ molecules in molecular junctions in (a), derived from Bader charge analysis. (d) PDOS for molecular junctions with distance *z* = 15.3 Å and *z* = 14.9 Å. (e) Spin-resolved electron transmission as a function of electron energy with respect to the Fermi level for the molecular junction, corresponding to the PDOS in (d). The PDOS and transmission spectral for the molecular junction at a closer distance *z* of 14.3 Å are shown in Figure S12.

The spin-resolved PDOS (Figure 4d) of Ni 3d (d_*yz*_, d_*xz*_) orbitals near the E_F_ shows a narrowing of the spin splitting and a slight broadening of the peaks as the tip approaches. The majority-spin PDOS shifts upward, and the spin-up and -down components move closer in energy, indicating a reduction of spin polarization. Correspondingly, the spin-resolved transmission spectra (Figure 4e) exhibit a similar trend, the spin-up and -down resonances (*T*↑(*E*) and *T*↓(*E*)) move closer to the *E*_F_, and the transmission at the Fermi level *T*(*E*_F_) increases. This behavior is consistent with a moderate enhancement of the molecule–substrate coupling. Together, these observations reveal increased charge screening from the electrodes and slightly increased molecule–substrate hybridization. The enhanced screening lowers the effective on-site Coulomb interaction *U*_eff_ and the exchange splitting, while the increased hybridization promotes partial charge redistribution between spin channels. As a result, the spin polarization in Ni 3d orbitals is reduced, driving the molecule toward a low-spin configuration. These results provide a consistent explanation for the experimentally observed quenching of the spin state. In addition, the atom PDOS (Figure S11) shows an increased overlap between the molecular states and the substrate (from both Br and Au atoms), further confirming the enhanced coupling to the substrate. This small increase in the coupling accounts for the slight peak broadening and the rise of *T*(*E*_F_). Overall, our data suggest that the Br-tip suppresses the molecular spin mainly through charge screening and coupling to the substrate of the frontier molecular orbitals near the Fermi level.

To assess the generality of Br-induced spin modulation, we performed analogous experiments with a CoCp_2_ molecule. CoCp_2_ adsorbed on Br-decorated Au(111) appears with a disk-like pattern (Figure 5a), corresponding to one Cp ring being exposed to vacuum. The d*I*/d*V* spectrum acquired with a metal tip above CoCp_2_ on the Br island is featureless at zero bias (Figure 5b), i.e., it lacks the Kondo resonance typically associated with a localized *S* = 1/2 spin state in CoCp_2_. While this absence of zero-bias resonance can in principle arise from very weak coupling, it is consistent with a quenching of the spin moment. We found that it was not possible to detach CoCp_2_ molecules from the Br island by vertical manipulation, which makes it challenging to directly investigate how varying the number of Br atoms influences the spin state of CoCp_2_. In contrast, CoCp_2_ molecules could be moved laterally on the surface by the tip. We found that the apparent height of CoCp_2_ remains essentially unchanged (390 pm) before and after lateral manipulation (Figure 5c,d) and was noticeably higher than the typical values observed for metallocenes.^36,37^ After manipulation along the indicated trajectory (red arrow), no Br atom was observed at the original adsorption site, as indicated by the dotted red circle. These observations suggest that Br remains bound to the CoCp_2_ molecule during manipulations, reflecting a relatively strong molecule–bromine interaction that likely plays a role in modifying the spin state. This can be further proven by comparing the d*I*/d*V* spectra before and after manipulation (Figure S13). In addition, systematic studies of CoCp_2_ on I clusters show that I interacts more weakly with CoCp_2_ but can still quench its spin state (Figure S14).

**Figure 5:**
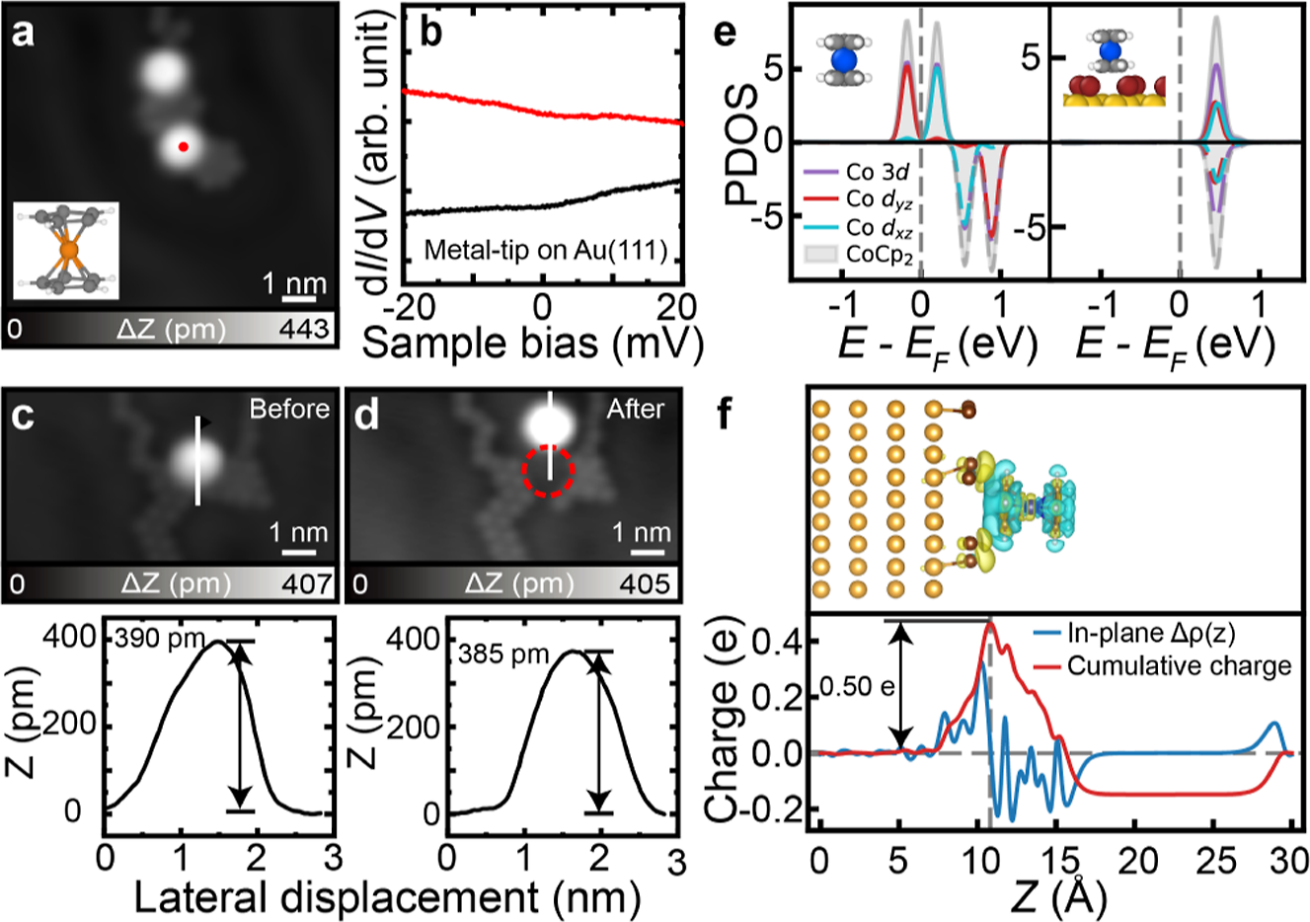
Magnetic property of CoCp_2_ molecules on a Br cluster on Au(111). (a) STM image of the CoCp_2_ molecule adsorbed on Br clusters. The inset shows the chemical structure of the CoCp_2_ molecule. (b) d*I*/d*V* spectrum recorded at the marked sites in (a) using a metal tip. The black curve is acquired on a clean Au(111) surface with a metal tip. (c, d) STM images captured before and after the manipulation of the CoCp_2_ molecule from the Br cluster, along with their corresponding line profiles shown in the lower panel. The line profiles were extracted along the white lines in the STM images, and the red arrow in (c) indicates the direction of manipulation. Scanning parameters: (a, c, d) *V* = 0.2 V, *I* = 10 pA; (b) *V*_ac_ = 0.5 mV. (e) PDOS of the gas-phase CoCp_2_ molecule and CoCp_2_ adsorbed on a Br island on Au(111). (f) The upper panel shows the side view of the CoCp_2_ on a Br island (9 atoms) on Au(111), showing charge transfer from the molecule to the Br island (isosurface at 0.0003e/Å^3^). The lower panel is the DFT-calculated in-plane electron density difference plot.

To support this interpretation, we carried out DFT calculations for CoCp_2_ adsorbed on a Br island. We did not consider Br clusters of varying sizes on Au(111), as experimental data on how the number of Br atoms affects the spin state of CoCp_2_ were not available. Planar-averaged charge density difference analysis shows an electron depletion of about 0.5 e from the molecule to the underlying Br atoms (Figure 5f), indicating substantial charge transfer. A comparison of the binding energy between NiCp_2_ (−1.14 eV) and CoCp_2_ (−2.14 eV) molecules on the Br island further supports the experimental observation of stronger binding for CoCp_2_. The computed PDOS exhibits only degenerate unoccupied states near the *E*_F_, in contrast to the singly occupied orbital in the gas-phase molecule. Correspondingly, the computed magnetic moment is 0.09 μ_B_, which is consistent with a fully quenched spin state. For comparison, the gas-phase CoCp_2_ molecule exhibits a magnetic moment of 1.0 μ_B_, corresponding to an *S* = 1/2 state. Therefore, the pronounced reduction in the spin moment upon adsorption confirms that Br-induced electron depletion effectively quenches the molecular spin, paralleling the behavior observed for NiCp_2_.

## Conclusions

In summary, we demonstrated that Br atoms serve as an effective means to tune the spin states of SMMs. For NiCp_2_, adsorption on Br-decorated Au(111) results in an electron depletion from the molecule, shifts one Ni 3d (d_*yz*_ and d_*xz*_) component above the Fermi level, and leaves one singly occupied state, thereby establishing an *S* = 1/2 state. Introducing a Br-tip further quenches the moment predominately via charge screening and increased the level of molecule–substrate hybridization. This precise control over the number of Br atoms enables reversible modulation between distinct spin states (*S* = 1, 1/2, and 0), highlighting the potential for dynamic and scalable spin control at the molecular level. The adsorption of CoCp_2_ on Br-decorated Au(111) exhibits a near-zero magnetic moment, confirming the generality of Br-driven spin quenching. These findings not only deepen our understanding of the interplay between the molecular electronic structure and magnetic behavior but also pave the way for the design of advanced spintronic devices where individual molecules can function as controllable quantum units.

## Methods

### STM Experiments

A homemade low-temperature STM operating at 4.3 K under ultrahigh vacuum conditions (*P* < 5 × 10^–10^ mbar) was used. The Au(111) substrate was repeatedly cleaned by cycles of Ar^+^-ion sputtering, followed by annealing at 700 K for 15 min. The sample temperature was monitored with both a pyrometer and a thermocouple. The tip was prepared by chemical etching of a thin W wire. Before the measurement, the tip apex was cleaned by repeatedly contacting the clean Au(111) surface, which consequently resulted in coverage by gold atoms. Leak valves were used to evaporate NiCp_2_ and CoCp_2_. Liquid bromine and solid iodine were introduced into glass tubes connected to UHV leak valves. Br and I atoms were then deposited onto the Au(111) surface through the leak valves while the surface was maintained at room temperature. The precursor pressures for Br_2_ and I_2_ deposition were 2 × 10^–8^ mbar and 5 × 10^–7^ mbar, respectively. STM images were recorded by using chemically etched tungsten tips.

### Theoretical Calculations

Spin-polarized DFT calculations were performed using the VASP package^38,39^ with the optB86 functional^40^ and the projector augmented wave method^41^ with an energy cutoff of 500 eV. The vacuum layer was larger than 10 Å ,and *k*-point sampling was only at the gamma point. The molecular adsorption was modeled on a periodic (*a* = *b* = 14.98 Å, γ = 60°) slab unit cell of the Au(111) with the bottom two layers kept fixed. To model the junction, we added another 4 × 4 Au surface unit cell holding the Br-tip and allowed the tip, molecules, and the top layer Au atoms to relax. All relaxed atoms were optimized until atomic forces were less than 0.02 eV/Å. Constant-height d*I*/d*V* maps were simulated using the PPSTM code^42,43^ with fixed tips and a pure *s*-wave orbital for a NiCp_2_ molecule on a Br island on Au(111).

The transport calculations were carried out using first-principles with a method based on nonequilibrium Green’s functions combined with DFT as implemented using the TranSIESTA package.^44^ By adding the VASP-optimized molecular junction region between two electrodes, we modeled with two surfaces, with one representing the substrate and another one holding the tip. Both electrodes were modeled with a 9-layer slab geometry of a surface unit cell *a* = *b* = 14.98 Å, γ = 60°. A double-ζ plus polarization (DZP) basis set was used to describe the NiCp_2_ molecule and surface-atom electrons. Diffuse orbitals were used to improve the surface electronic description and a single-ζ plus polarization basis set for the Au electrodes. The *k*-point sampling was 5 × 5 for the transmission calculations.

## Supplementary Material



## Notes

Line profiles of NiCp_2_ molecules on a Br island; a series of manipulation experiments; STM images of Br atoms at both low and high coverages; adsorption geometries of a NiCp_2_ molecule on a Br island; spin-resolved PDOS of NiCp_2_; Bader charge distribution; atom PDOS; and calculated magnetic moments of the NiCp_2_ molecule (PDF)
