# Supplementary material for: Br-Mediated Spin-State Control in Nickelocene and Cobaltocene
Source: J Am Chem Soc. 2026 Jan 10;148(3):3356–64. doi: 10.1021/jacs.5c17873 (PMC12856892; doi:10.1021/jacs.5c17873)
Supplement: Supplementary file 1 [file ja5c17873_si_001.pdf]

Br-Mediated Spin-State Control in Nickelocene and Cobaltocene

Donglin Li[1], Nan Cao[2], Adam S. Foster[2,3]*, and Shigeki Kawai[1,4]*

[1]Center for Basic Research on Materials, National Institute for Materials Science, 1-2-1 Segen, Tsukuba, Ibaraki 305-0047, Japan.

[2]Department of Applied Physics, Aalto University, Espoo 11100, Finland.

[3]Nano Life Science Institute (WPI-NanoLSI), Kanazawa University, Kakuma-machi, Kanazawa, Ishikawa 920-1192, Japan.

[4]Graduate School of Pure and Applied Sciences, University of Tsukuba, 1-1-1 Tenodai, Tsukuba, Ibaraki 305-8571, Japan.

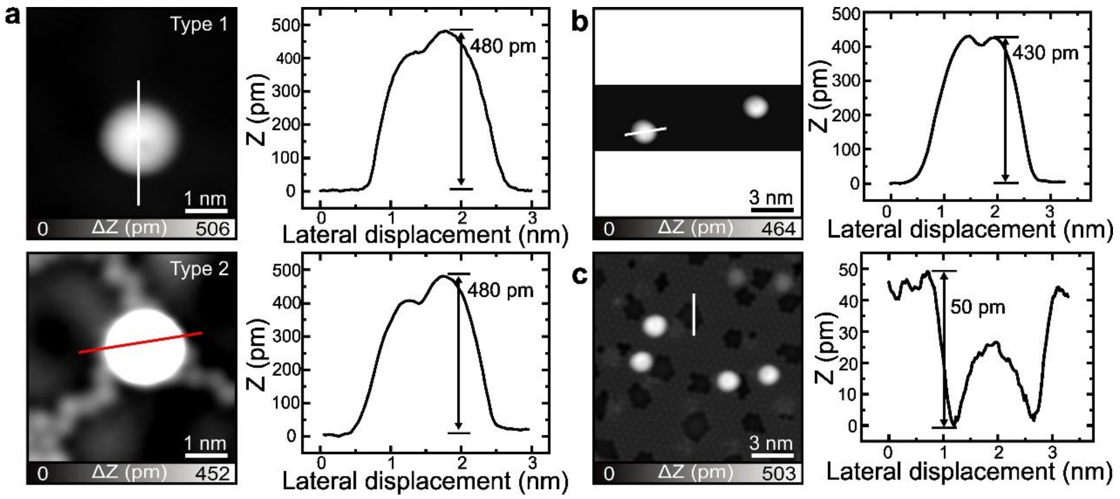

Figure S1. STM images of $NiCp_2$ molecules adsorbed on (a) Br clusters, (b) bare Au(111) surface, and (c) Br island are shown alongside their corresponding line profiles, which were extracted along the white and red lines in the images. Scanning parameters: $V = 0.2$ V, $I = 10$ pA.

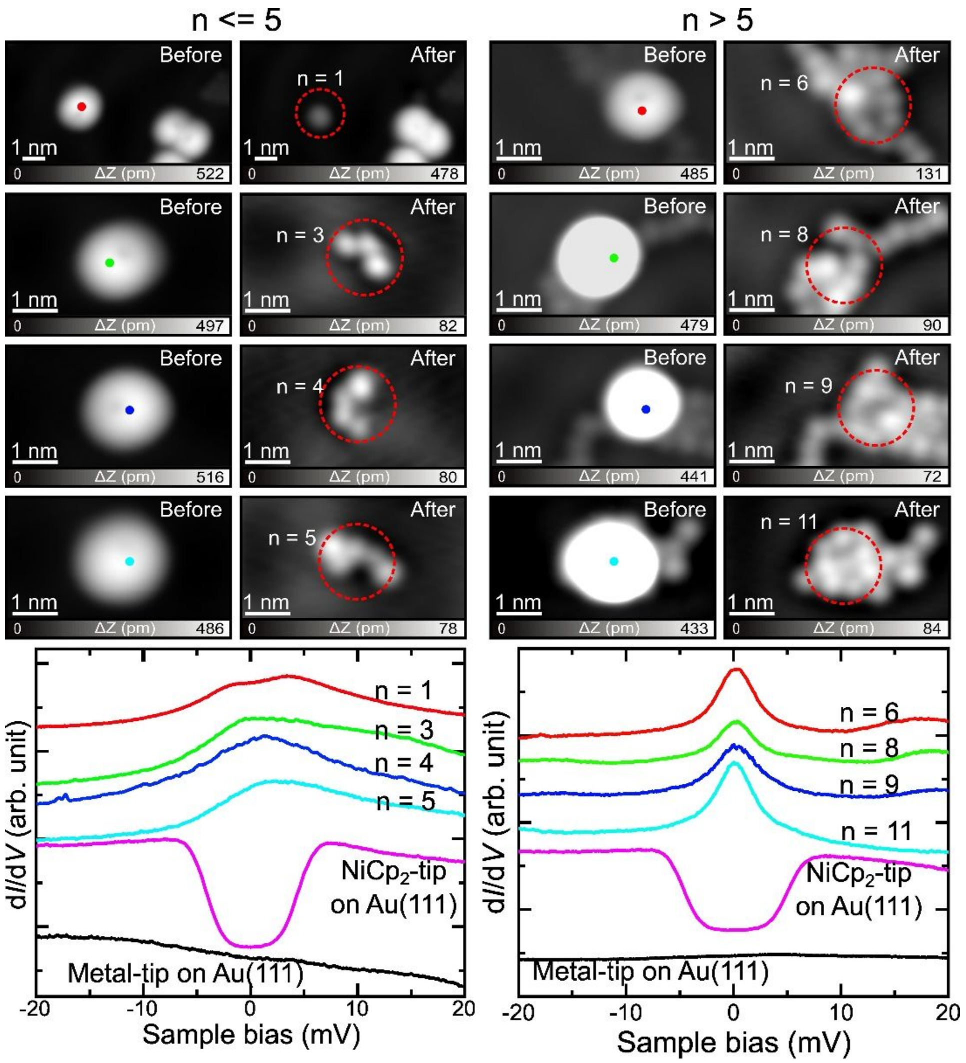

Figure S2. A series of STM images captured before and after picking up $NiCp_2$ molecules adsorbed on Br clusters, illustrating the number of Br atoms beneath $NiCp_2$. The dI/dV spectra recorded at the marked points in the STM images. The green and black curves were obtained over the Br island using a $NiCp_2$ tip and a metal tip, respectively. Scanning parameters: $V = 0.2$ V, $I = 10$ pA.

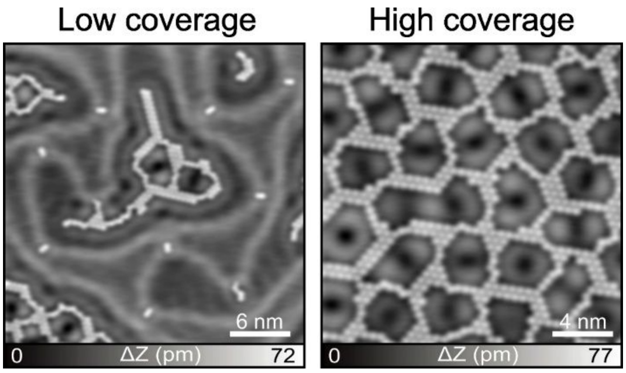

Figure S3. STM images of Br atoms at both low and high coverages. Scanning parameters: $V = 200$ mV, $I = 10$ pA.

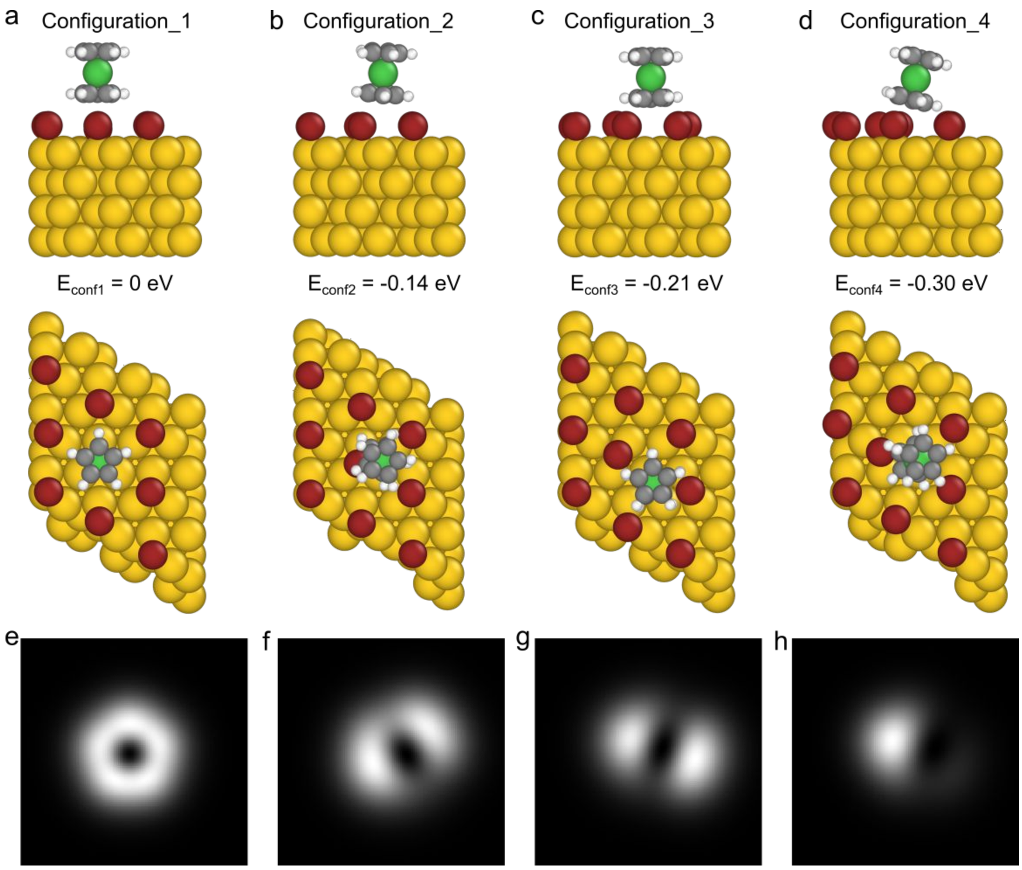

Figure S4. Adsorption geometries and simulated STM images of a $NiCp_2$ molecule on a Br island on Au(111) surface. (a)-(d) Optimized adsorption configurations (Configuration_1 to Configuration_4) with relative adsorption energies E_{conf} are presented. The Br island was constructed with a $(\sqrt{3} \times \sqrt{3})R30°$ configuration on Au(111), reflecting the adsorption geometry revealed in the experiments. These four configurations were generated based on distinct local environments of the Br island, including adsorptions on top of a Br atom (conf1), at the edge of a Br atom (conf2), on the bridge site between two Br atoms (conf3) and on the hollow region between three Br atoms (conf4). The computed molecular magnetic moment for the above adsorption configurations is shown in Table 2. (e)-(h) Simulated constant-height dI/dV images acquired with metallic tip (image sizes: 12 × 12 Å^2) at 0.2 eV, corresponding to the configuration in (a)-(d). We note that conf2 most closely reproduces the experimental asymmetric dI/dV feature, although not being the lowest energy structure. This indicates that the metastable adsorption geometry can be preferentially stabilized in experimental conditions.

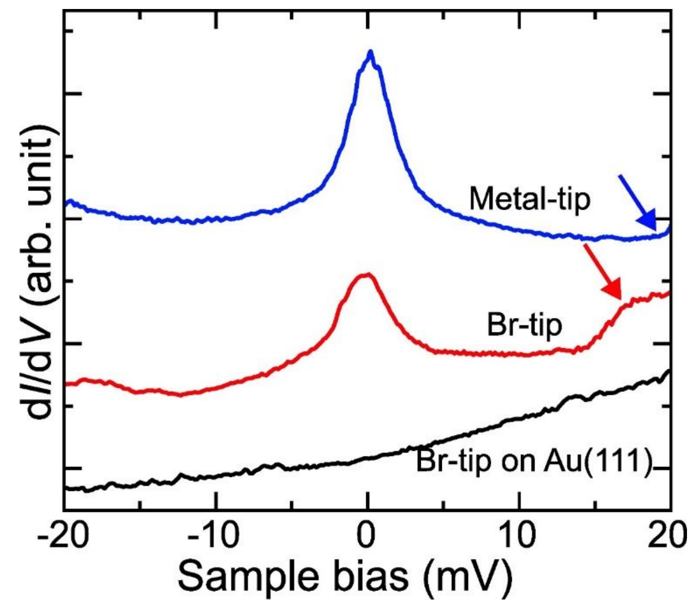

Figure S5. The d*I*/d*V* spectra extracted from Figures 2f and 2g for better comparison. With the Br-terminated tip, the Kondo resonance is relatively weaker than that obtained with the metal tip, making the vibrational side peak (indicated by red arrow) more pronounced. In addition, as both the intensity and position of the side peaks vary depending on the adsorption site (Figure 2b), the tip condition may also influence the observed vibrational energy. The side peak may shift beyond 20 mV, leaving only a small portion (indicated by blue arrow) visible within the measured range. The black curves are acquired on a clean Au(111) surface with the Br-tip.

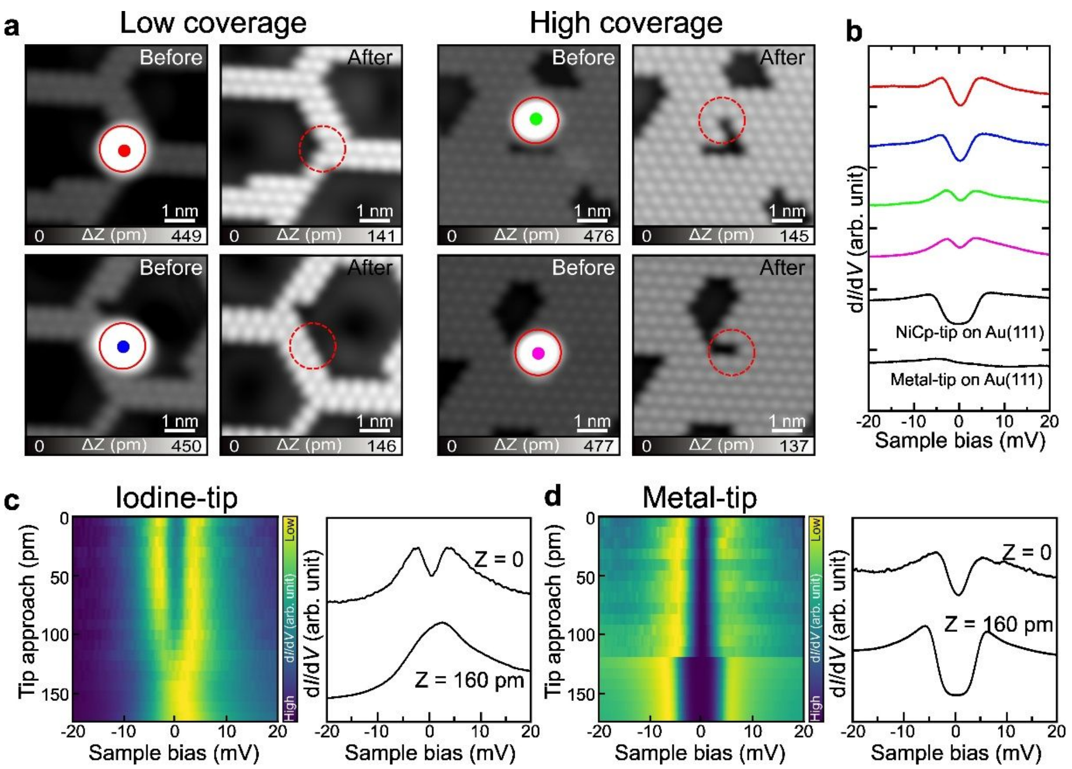

Figure S6. Effect of I atoms on the spin state of a $NiCp_2$ molecule. (**a**) STM images of $NiCp_2$ molecules adsorbed on low- and high-coverage I islands before and after the molecular manipulation. The dotted red circle indicates its original position. (**b**) d*I*/d*V* spectra recorded at the marked sites in (a) using a metal-tip. The black curves are acquired on a clean Au(111) surface with a $NiCp_2$-tip and metal-tip, respectively. (**c**) Two-dimensional intensity plots of d*I*/d*V* spectra acquired on the $NiCp_2$ with a I-tip (**d**) and a metal-tip (g) at 170 pm different tip-sample distances. The intensities have been normalized. The initial setpoint was $V = 20$ mV, $I = 500$ pA. Measurement parameters: (a) $V = 0.2$ V, $I = 10$ pA; (b, c, d) $V_{ac} = 1$ mV.

Systematic studies using iodine atoms were also performed to investigate their influence on the $NiCp_2$ molecule. As shown in Figure S6a, both low and high coverages of iodine were investigated. Similar to the case of $NiCp_2$ molecules on the Br island, individual $NiCp_2$ molecules can also be picked up from the I island. After manipulation, the presence of I atoms underneath the molecules can be confirmed. Unlike $NiCp_2$ adsorbed on Br atoms, the d*I*/d*V* spectra recorded over $NiCp_2$ molecules on I atoms (Figure S6b) exhibit dip features with a gap significantly smaller than the intrinsic gap of $NiCp_2$. Notably, $NiCp_2$ molecules on the high-coverage I island show a narrower gap compared to those on the low-coverage island, indicating that the spin excitation energy of $NiCp_2$ decreases with increasing numbers of I atoms. To further modulate the spin state of $NiCp_2$, we performed height-dependent STS measurements on the $NiCp_2$ molecule adsorbed on the I island using an I-terminated tip (Figure S6c). Interestingly, as the tip approached, the dip feature gradually disappeared, and a broad peak feature emerged. Compared with the same experiment on the Br island, this behavior suggests that the lower electronegativity of iodine results in a weaker ability to tune the spin state

of $NiCp_2$ molecules. In addition, height-dependent STS measurements performed with a metal tip (Figure S6d) showed that the $NiCp_2$ molecule was picked up by the tip as it approached. This behavior was not observed for $NiCp_2$ on the Br island, further supporting the conclusion that the interaction between I atoms and $NiCp_2$ molecules is weaker than that between Br and $NiCp_2$.

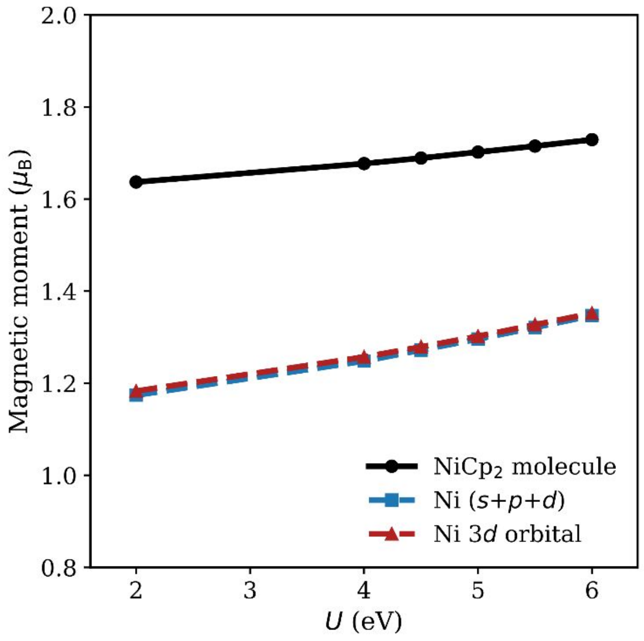

Figure S7. Calculated magnetic moments of $NiCp_2$ molecule as a function of the on-site Coulomb interaction *U*. The total magnetic moment of the molecule (black), the local moment in the Ni atom (blue) and the Ni 3*d* orbital (red) all increase slightly with increasing *U*, indicating that stronger on-site correlation enhances the spin polarization on the molecule. The overall variation is not pronounced, suggesting that the spin state of the $NiCp_2$ molecule is robust with respect to the choices of *U*. Thus, no additional *U* correction was applied in this work.

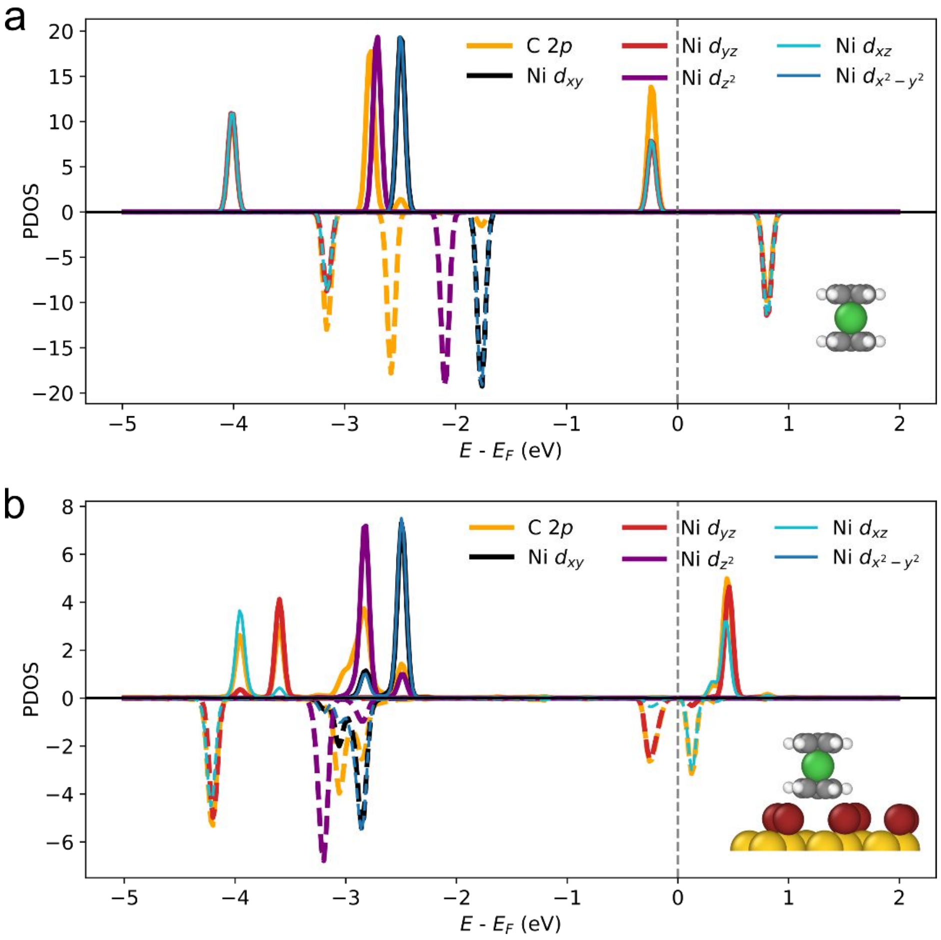

Figure S8. Spin-resolved PDOS of the $NiCp_2$ molecule in (a) gas phase and (b) absorbed on the Br island on Au(111). In the gas phase $NiCp_2$ molecule, the 3*d* orbitals of Ni present two completely degenerate d_{xz} and d_{yz} orbitals near the Fermi level, while their majority-spin PDOS distribute below the Fermi level and their minority-spin PDOS distribute above the Fermi level. This indicates that the d_{xz} and d_{yz} orbitals are not fully occupied. Together with the *p* orbitals of Cp rings, displaying a big overlap with these two orbitals, hence they contribute dominantly to the total magnetic moments of 1.7 μB and result in a spin S = 1 state from our calculations. Upon adsorption, the degenerate d_{xz} and d_{yz} orbitals split with the $3d_{xz}$ orbital move above the Fermi level (unoccupied), $3d_{yz}$ still lie below the Fermi level (occupied), resulting in a singly occupied state, consistent with a spin S = 1/2 state.

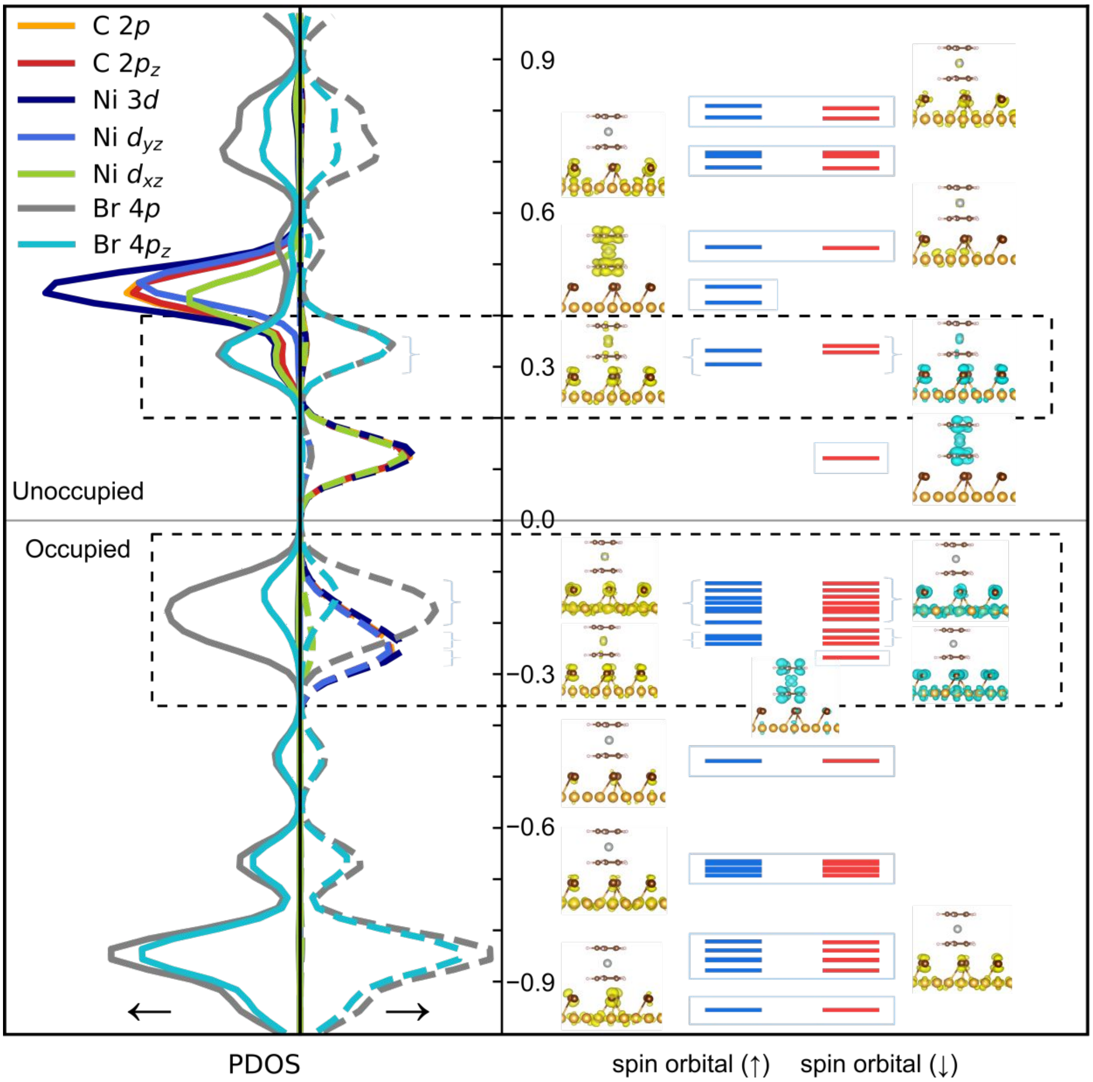

Figure S9. Spin-resolved PDOS and molecular orbitals for $NiCp_2$ molecule absorbed on the Br island on Au(111). The left panel shows spin-resolved PDOS projected onto selected C2p, Ni 3d and Br 4p orbitals. Within the energy windows indicated by the dashed rectangles (0.2 to 0.4 eV and -0.15 to -0.35 eV), the PDOS shows contributions from C 2p*z*, Ni 3d (d*xz* and d*yz*) orbitals and Br 4p (p*z*) orbitals, indicating the $NiCp_2$ and Br hybrid states. The corresponding molecular orbitals (right panel, isosurface at 0.015-0.003 e/Å^3) shows spatial distributions delocalized over the Ni center, Cp rings, and the Br layer, consistent with these hybrid states. The spin up and down channels show different energies and spatial weights within the same energy windows. For example, between 0.2 and 0.4 eV, the spin up hybrid states exhibit stronger contribution on Br 4p, while the spin down states becomes even more localized on Br. This asymmetric hybridization indicates the spin density transition from $NiCp_2$ to Br atoms, consistent with the observed charge redistribution and the reduction of magnetic moment of the $NiCp_2$.

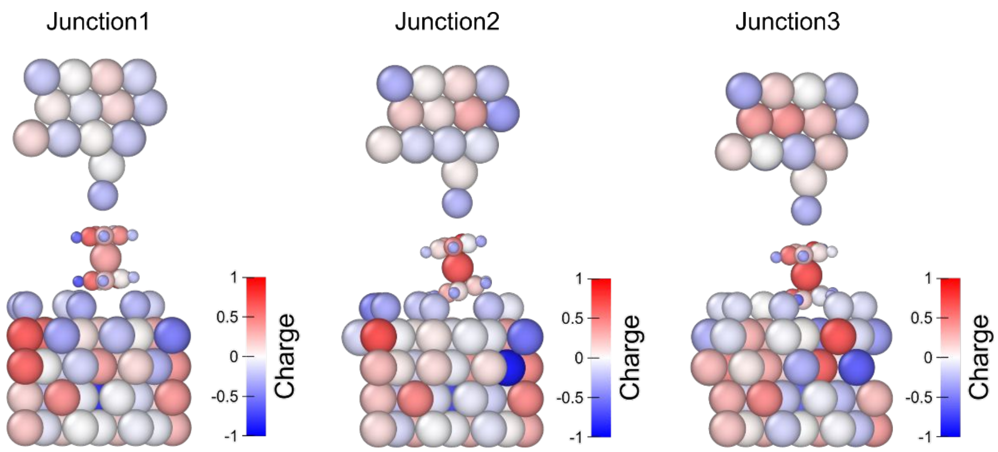

Figure S10. Bader charge distribution of three molecular junctions at different tip-substrate distances. The total Bader charges of the molecule, Br-tip, Br island and Au substrate are summarized in Table 3.

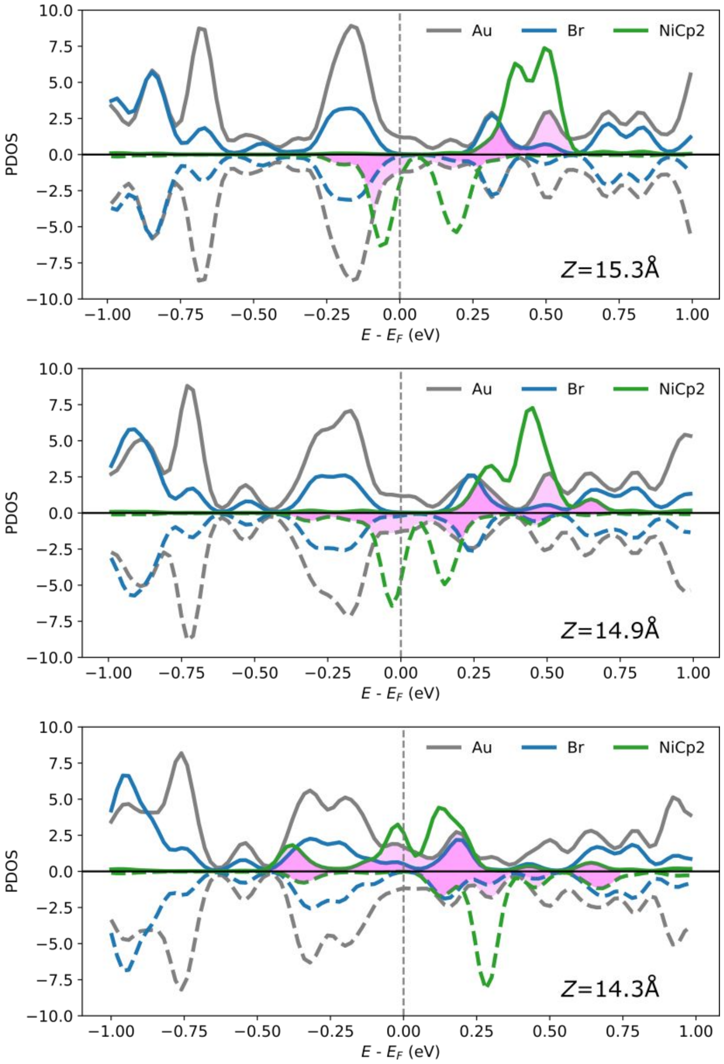

Figure S11. Atom-projected PDOS of the $NiCp_2$ molecule, Br and Au atoms in the molecular junction at three different distances. The magenta region highlights the energy intervals near the Fermi level where the PDOS of $NiCp_2$ overlaps with that of Br/Au substrate, indicating a direct visual measure of the molecule-substrate hybridization. As the distance decreases from z = 15.3 Å to 14.9 Å, the $NiCp_2$ PDOS peaks (green) in 0.25-0.6 eV energy range becomes broader and shift toward the Br/Au PDOS peaks (blue and gray). This results in a visibly enlarged energy interval (0.1-0.75 eV at 14.9 Å) where the $NiCp_2$ and substrate PDOS overlap. This progressive broadening and energy shift indicate an enhanced hybridization with decreasing tip distance. Consistently, the exchange splitting of $NiCp_2$ frontier states is reduced, further indicating an increase of molecule-substrate hybridization. This trend is accompanied by a slight charge rearrangement in the junction (Table S4), and a moderate decrease of the magnetic moment (Figure 4b). At the shorter distance at z = 14.3 Å, the overlap becomes significantly larger and the exchange splitting of frontier states is further reduced, consistent with a much stronger coupling to the substrate within a compressed junction geometry.

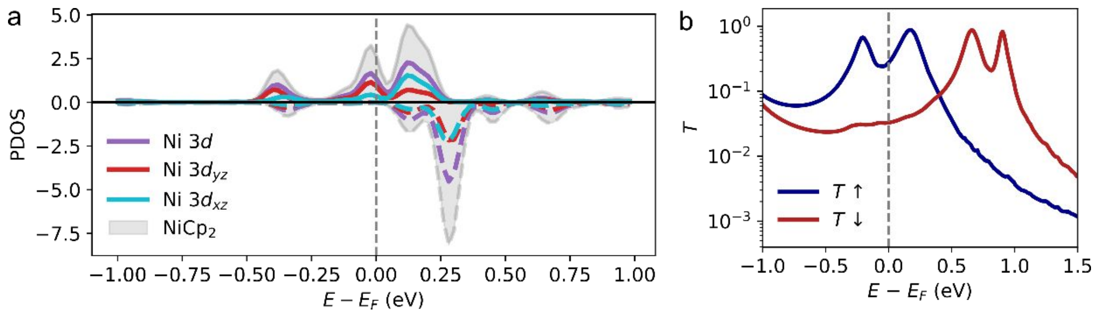

Figure S12. PDOS and transmission of the molecular junction at distance z = 14.3 Å. (a) Density of states projected on the Ni 3d, 3d_{yz}, 3d_{xz} orbitals and the $NiCp_2$ molecule. (b) Spin-resolved electron transmission as a function of electron energy with respect to the Fermi level. The clear correspondence between spin up and spin down peaks in the transmission function and the PDOS enables assignment of both peaks to the transmission through the spin-polarized, nearly degenerate frontier molecular orbitals of the molecule. The increased broadening of the PDOS signal and transmission features compared with those in Figures 4d and 4e, indicates stronger molecule-substrate hybridization and reduced exchange splitting at smaller distance.

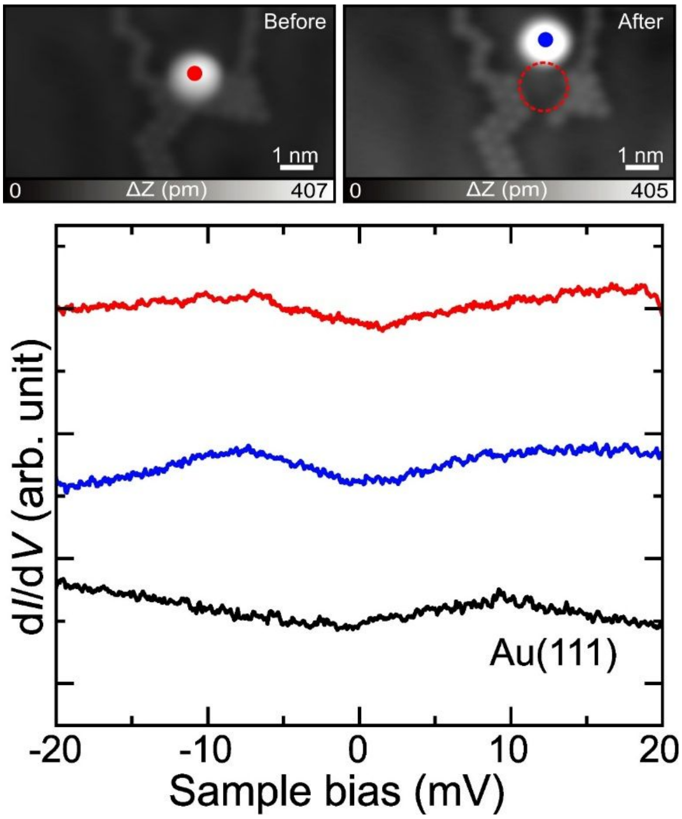

Figure S13. dI/dV spectra acquired before (red curve) and after (blue curve) manipulation of a $CoCp_2$ molecule on the Br island. The black curve is acquired on a clean Au(111) surface with a metal tip.

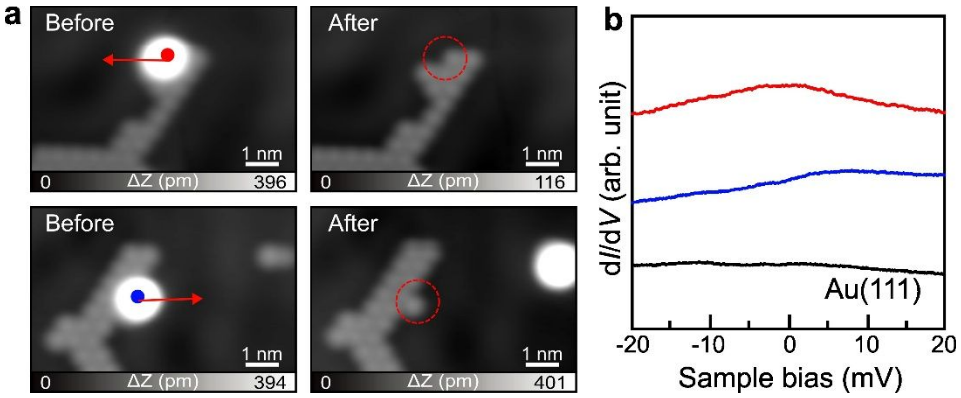

Figure S14. Magnetic property of $CoCp_2$ molecules on I cluster on Au(111). (a) STM images captured before and after the manipulation of the $CoCp_2$ molecule from I cluster. The dotted red circle indicates its original position. (b) dI/dV spectrum recorded at the marked sites in (a) using a metal tip. The black curve is acquired on a clean Au(111) surface with a metal tip. Scanning parameters: (a) V = 0.2 V, I = 10 pA. (b) V_{ac} = 1 mV.

Table S1. Magnetic moments of the $NiCp_2$ molecule calculated using different exchange-correlation functionals. The similar values obtained from semilocal (DFTD3, OPTB86) and hybrid functionals (PBE0, HSE06) confirm that all functionals capture the essential spin polarization reasonably accurately. Therefore, OPTB86 was selected for all calculations in this work.

Functional	$NiCp_2$ Mag (μ_B)	Ni atom Mag (μ_B)	Ni 3d Mag (μ_B)
DFTD3	1.61	1.10	1.12
OPTB86	1.60	1.10	1.11
PBE0	1.70	1.31	1.33
HES06	1.70	1.30	1.32

Table S2. Comparison of molecular charge and magnetic moment for $NiCp_2$ and $CoCp_2$ molecule on the Br island/Au(111) surface obtained from DFT+U calculations (U = 3 and 5 eV).

As shown below, both the molecular charge and magnetic moment of $NiCp_2$ and $CoCp_2$ molecule on the Br island/Au(111) show only minor variations with the used Hubbard U (3 and 5 eV). For $NiCp_2$, the magnetic moment remains around 1 μ_B and the charge transfer slightly decreases with increasing U, indicating a robust and localized spin on the Ni. For $CoCp_2$, a charge transfer of ~0.5 e from the molecule to the Br layer lead s to a fully quenched magnetic moment, which is independent of U. These results confirm that the observed spin reduction mainly originates from charge transfer, while the inclusion of U only weakly influences both the charge and spin states.

Molecule	U (eV)	Molecular charge (e)	Molecular Mag (μ_B)	Metal atom Mag (μ_B)	Ni 3d Mag (μ_B)
$NiCp_2$	3	0.37	0.98	0.71	0.72
	5	0.32	1.10	0.88	0.88
$CoCp_2$	3	0.53	0	0	0
	5	0.53	-0.002	-0.002	-0.002

Table S3. Computed magnetic moments of the $NiCp_2$ molecule in four different adsorption configurations on the Br island on Au(111) shown in Figure S3. The table presents the total magnetic moment of the molecule, the Ni atom, and the contribution from the Ni 3*d* orbitals. The results show that the magnetic moments of different adsorption configurations are nearly identical in magnitude. The opposite sign reflects opposite spin orientations. This indicates that the adsorption geometry has little effect on the spin polarization of the molecule.

Adsorption configuration	$NiCp_2$ Mag (μ_B)	Ni atom Mag (μ_B)	Ni 3d Mag (μ_B)
conf1	0.87	0.56	0.57
conf2	-0.95	-0.62	-0.63
conf3	-0.87	-0.56	-0.57
conf4	-0.90	-0.58	-0.59

Table S4. Computed charge quantity of $NiCp_2$ molecule, Br-tip, Br island and Au substrate in three junctions shown in Figure S9.

Junction	Distance (Å)	$NiCp_2$ charge (e)	Br-tip charge (e)	Br island (e)	Au substrate (e)
1	15.30	0.38	-0.14	-1.94	1.70
2	14.90	0.23	-0.15	-1.91	1.83
3	14.30	-0.07	-0.14	-1.03	1.24

The charge of the Br island is essentially unchanged in the experimentally accessible distance regime (Junction1 and Junction2), and Junction3 corresponds to a compressed geometry included to illustrate the limiting case. In this configuration, the apparent large change in the Br charge arises from substantial geometry distortion and the resulting shift of the Bader partition boundaries, rather than a chemical change of the Br layer.
